# Survival and Growth of *Vibrio cholerae*, *Escherichia coli*, and *Salmonella* Spp. in Well Water Used for Drinking Purposes in Garoua (North Cameroon)

**DOI:** 10.1155/2013/127179

**Published:** 2013-08-07

**Authors:** Moussa Djaouda, Bouba Gaké, Daniel Ebang Menye, Serge Hubert Zébazé Togouet, Moïse Nola, Thomas Njiné

**Affiliations:** ^1^University of Maroua, Higher Teachers' Training College, P.O. Box 55, Maroua, Cameroon; ^2^University of Yaoundé I, Laboratory of General Biology, P.O. Box 812, Yaoundé, Cameroon; ^3^Centre Pasteur du Cameroun, Annexe de Garoua, B.P. 921, Garoua, Cameroon

## Abstract

The ability of strains of faecal bacteria (*Vibrio cholerae*, *Escherichia coli* ATCC 25922, and four strains of *Salmonella* isolated, resp., from well water, pig, poultry, and human urine in Garoua) to survive or grow in well water microcosms was compared. Water samples were obtained from two wells in Garoua (north Cameroun). Autoclaving at 121°C for 15 min and filtration through 0.2 µm filter were used to make microcosms. Microcosms were constituted of unfiltered-autoclaved, filtered-nonautoclaved, and filtered-autoclaved well waters. Bacterial strains were inoculated at initial cell concentration of 3 Log_10_CFU/mL. All strains were able to survive/grow in used microcosms, and a maximal concentration of 5.61 Log_10_CFU/mL was observed. Survival abilities were strain and microcosm dependent. The declines were more pronounced in filtered-nonautoclaved water than in the other microcosms. *E. coli* and *Salmonella* sp. (poultry strain) lowered to undetectable levels (<1 Log_10_CFU/mL) after two days of water storage. *V. cholera* decreased over time, but surviving cells persisted for longer period in filtered-nonautoclaved water from well W1 (1.91 Log_10_CFU/mL) and well W2 (2.09 Log_10_CFU/mL). Competition for nutrients and/or thermolabile antimicrobial substances synthesized by “ultramicrocells” or by the autochthonous bacteria retained by the filter might affect the bacterial survival.

## 1. Introduction 

Diarrheal diseases are among the major causes of mortality in developing countries. Most cases of diarrhea (88%) are attributable to unsafe water and inadequate sanitation and hygiene [1]. According to Ngwe and Banza-Nsungu [2], diarrheal diseases (usually linked to faecal contamination of water) cause annually 15% to 20% of all deaths in Cameroon. Cholera outbreaks are almost endemic in the norther part of this country. Cameroon Red Cross and International Federation of Red Cross and Red Crescent Societies stated that cholera outbreak which occurred in Cameroon in 2010 affected about 10,741 people and killed 650. The northern part of Cameroon and the littoral region were the most affected localities with 9,406 cases and 600 deaths in the far north region, 511 cases and 24 deaths in the north region, and 457 cases and 12 deaths in the littoral region.

Groundwater from wells, springs, and boreholes is largely consumed due to limited tap water supply in Cameroon. These water sources have poor hygienic quality due to their proximity to latrines. Protection and upgrading of wells were recommended for safe drinking and domestic water sources [3]. Usually, groundwater is stored in containers at home for different household uses.

Because faecal bacteria are distributed widely in point-of-use water sampled from storage vessels among well water consumers in Garoua [3], there is a considerable need for implementing water treatment at home. Consequently, it is important to develop and implement point-of-use water disinfection methods that can be used to improve water quality at the household level.

Household water treatment results in clean water and one usually devoid of coliforms. Filtration with small-pores filters and boiling have been shown to be effective at removing many microbes and suspended solids [1]. Well water can be physically disinfected by boiling, filtration, or combination of the two techniques.

However, unhygienic handling of water during transport or within home can contaminate even previously safe water [1, 4]. In case of secondary contamination, within households, pathogenic bacteria introduced into the treated water could have different destiny according to disinfection technique used. During the period of storage, contamination by consumers and aftergrowth may lead to high levels of faecal bacteria in treated well water, thus posing a risk for consumers [5]. Treated or not, well water used for drinking purposes could then be a vector of many waterborne diseases.

Nola et al. [6] found that storage of untreated groundwater for a long period would increase the health risk to the consumers in the short term if it contains potentially pathogenic bacteria due to their potential growth and activity. Solutes in water can be used as sources of nutrients for bacterial populations and could therefore markedly enhance bacterial growth [7]. The versatile character of bacteria allows them to adapt to numerous apparently hostile conditions as lower temperatures [6].

Stored water is one of the most important types of water supplies for domestic purposes in Cameroon. The majority of studies undertaken on water in Cameroon has focused on surface and groundwater quality with little work being devoted to point-of-use water quality assessment.

Information about survival/growth capacity of pathogenic bacteria in treated well water stored under local climatic condition is necessary to advice consumers about correct handling of well-treated well water at home.

This research aimed at evaluating survival and growth of some faecal and pathogenic bacteria in autoclaved and/or filtered well water used for drinking purposes in Garoua region. Our study explored the evolution of the abundances of some faecal bacteria in the well water, treated by autoclaving and/or filtration, during its storage in household conditions.

## 2. Materials and Methods

### 2.1. Study Site

The study was carried out in Garoua (north Cameroon). This region is located at latitude 9°18′N and longitude 13°24′E. The Garoua region is made up of heterogenic soils [8]. It is characterized by a tropical climate with two seasons: a long dry season (October to April) and a short rainy season (May to September) [9]. Generally, rainfalls do not exceed 985 mm in the year. The monthly average temperature varies from 18°C to 42°C; the highest value is often noted during March and the lowest in January [9].

Two well water points (W1, W2) were selected based on their highest importance as a drinking water supply, significant difference between their mineralization levels [3], and the permanent presence of water in these wells during all seasons of the year.

### 2.2. Water Samples Collection

Four water samples (*n* = 4) were collected bimonthly at each sampling point during dry season (February-March). The water collection method replicated the community members water extraction method, using bucket.

At each site (W1, W2), water samples were collected in 500 mL sterile glass bottles, respectively, coded in three replicates and in corresponding 100 mL clean polyethylene bottles. Samples collected in polyethylene bottles were used for physicochemical analysis at the sample site. Those collected in glass bottles were destined for bacteriological analyses and experiments in laboratory.

### 2.3. Physicochemical Analysis of Well Water

The main physicochemical parameters considered were temperature, total dissolved solids (TDS), pH, electrical conductivity (EC), and salinity. These parameters were chosen in accordance with their general importance on bacterial metabolism and the availability of our laboratory equipments. Physicochemical analyses were made directly in the field using the techniques described by Rodier [10].

### 2.4. Bacteriological Analysis of Well Water

Heterotrophic bacteria were enumerated using the spread plate method with Plate Count Agar (Bio-Rad, France), incubated at 37°C for 72 hours. Membrane filtration was used to enumerate qualitative microbial indicators (total coliforms, faecal coliforms, *Escherichia coli,* and faecal streptococci) according to the standard methods [11]. The m-Endo LES (Difco Laboratories, Detroit, MI, USA) agar was used for the enumeration of total coliforms, faecal coliforms and *E. coli*. Slanetz-Bartley and Bile Esculin Azide (BEA) agars (Biokar Diagnostics, Beauvais, France) were used for faecal streptococci counts [12]. All analyses were done out in triplicate.

### 2.5. Water Microcosms

For each sample site, one of the 3-bottled samples replicates was analyzed for heterotrophic bacteria and qualitative microbial indicators as previously indicated. The second was autoclaved at 121°C for 15 min to destroy biological and heat sensitive antimicrobial agents. The third replicate was filtered and divided into two water samples: one was autoclaved, to destroy biological and heat sensitive antimicrobial agents that might pass through the 0.2 *μ*m filters (Millipore Corporation, Bedford, MA 01730, USA), and the second was not. The microbiological quality control of all experimental waters was realized; none of the bacteria used in this experiment was found.

### 2.6. Test Bacterial Strains

Three bacterial species were used for their importance to the environment and public health: *Vibrio cholerae, E. coli, and Salmonella* sp. Strains of *V. cholerae* (O1 Ogawa, El Tor) and *E. coli* ATCC 25922 were obtained from the Centre Pasteur of Cameroon and four strains of *Salmonella* sp. were isolated, respectively, from well water, pig, poultry, and human urine in Garoua. Their biochemical identification was performed according to Holt et al. (2000) procedures.

### 2.7. Preparation of Bacterial Stocks

For the preparation of bacterial stocks, a colony forming unit (CFU) of each strain from standard agar medium was inoculated into 100 mL of nutrient broth for 24 h at 37°C. The strain of *V. cholerae* was grown on alkaline nutrient agar and each of the other strains on standard nonselective Plate Count Agar (Bio-Rad) for later use.

Cells were then harvested by centrifugation at 3000 g for 10 min at ambient temperature and washed twice with sterile NaCl solution (8.5 g/L). 

### 2.8. Inoculation of Microcosms

The washed cells were suspended in autoclaved filtered water from each well (W1 and W2). From these new solutions, inoculations in three flasks (filtered-nonautoclaved water, filtered-autoclaved water, and unfiltered-autoclaved water) were performed for each well. Based on our preliminary study, cell concentration was adjusted at 10^3^ CFU/mL. Only one bacteria strain was added in each solution in the flask. Flasks were then incubated without shaking, in the dark, at room temperature (30 ± 2°C). The containers used by population in Garoua allow a time of well water storage up to three days and most households store water for 24 h. For this reason, our study used one day as observation time unit. The water storage lasts for three days and analyses were performed after 24 h, 48 h, and 72 h to determine bacterial abundances. Experiments were done thrice.

### 2.9. Growth and Survival of Test Bacterial Strains in Water Microcosms

Bacterial abundances were determined immediately after inoculation, at regular intervals (one day) up to three days by the spread plate procedure. Samples were serially diluted in a 0.85% (w/v) NaCl solution. The strain of *V. cholerae* was grown on TCBS agar (Merck KGaA, Darmstadt, Germany) and each of the other strains on standard nonselective Plate Count Agar (Bio-Rad). All analyses were performed according to the APHA [11] procedures.

Bacterial abundances are expressed as colony forming unit (CFU) per volume of water. The number of colony forming units (CFU) is multiplied by the dilution factor and expressed in CFU/mL of water.

### 2.10. Data Analysis

The values of bacterial counts (*x*) are transformed using the equation *y* = Log_10_(*x* + 1). The arithmetic mean of the log-transformed (base 10) values was utilized to summarize bacterial abundance at a given time.

## 3. Results and Discussion

### 3.1. Results

#### 3.1.1. Physicochemical Characteristics of Well Water

The results of the physicochemical analysis show that water samples range from acidic to basic with low to high mineralization. The TDS and salinity of water samples also varied with water source.

The well W2 had the highest pH value, a relatively higher salinity, TDS, and also the highest electrical conductivity (Table 1). The variation of water properties by different types of treatment was characterized by strong variations in pH, conductivity, TDS, and salinity.

Although water samples of different microcosms did not have the same physicochemical characteristics as the well they have been taken from, they followed the tendency of the parent points. Filtration and autoclaving both reduced the conductivity, TDS, and salinity levels of the well water samples. These treatments enhanced the water pH.

#### 3.1.2. Bacteriological Quality of Well Water

Abundances of each bacterial group isolated from sampled wells are presented in Table 2. They indicate clear evidence of faecal water contamination. The concentrations of total coliforms, faecal coliforms, heterotrophic bacteria, and faecal streptococci varied from one well to another, with the highest values recorded at well W2 (Table 2). However, *E. coli* were more represented in well W1 water samples. The water samples derived from used microcosms were devoid of all the previous microorganisms.

#### 3.1.3. Survival of Bacterial Agents in Well Water

**Figure 1:**
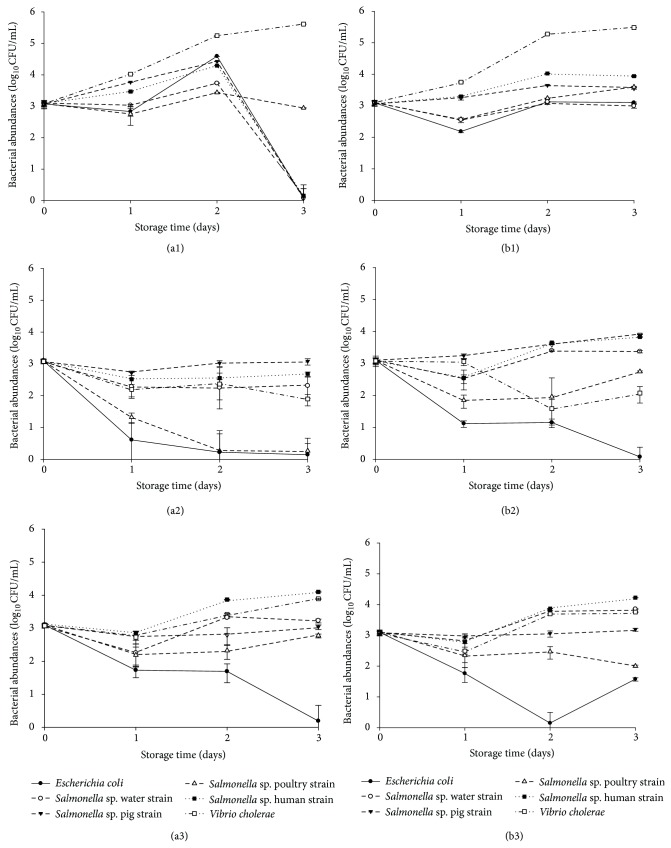
Survival curves of bacterial strains in well W1 water, unfiltered-autoclaved (a1); filtered-nonautoclaved (a2); filtered-autoclaved (a3), and well W2 water, unfiltered-autoclaved (b1); filtered-nonautoclaved (b2); filtered-autoclaved (b3).

The test bacteria were added at concentration 3 Log_10_CFU/mL. The viable counts of the six test bacteria increased after one day in unfiltered-autoclaved water, but the decrease depended on the organism and the water sample in which they were examined. The population of *E. coli* and *Salmonella* sp. decreased after two days in unfiltered-autoclaved water derived from well W1. In unfiltered-autoclaved water derived from well W2, after an initial decrease or latency phase, the viable counts increased and remained almost constant during the experimental period (Figure 1). Abundances of *V. cholerae* in unfiltered-autoclaved water (4.04 to 5.61 Log_10_CFU/mL in W1 microcosm; 3.78 to 5.49 Log_10_CFU/mL in W2 microcosm) were higher than those of other bacteria over the whole incubation period.

Using filtered-nonautoclaved water from well W1, *E. coli* and *Salmonella* sp. (Poultry strain) lowered to undetectable levels (<1 Log_10_CFU/mL) after two days of water storage (Figure 1). After the first day decrease, *Salmonella* sp. (pig strain), *Salmonella* sp. (water strain), and *Salmonella* sp. (human strain) grew or maintained their numbers throughout the water storage period. *Salmonella* sp. (pig strain) population reached 3.07 Log_10_CFU/mL in well W1 microcosm and 3.90 Log_10_CFU/mL in well W2 microcosm while *Salmonella* sp. (human strain) reached 2.70 Log_10_CFU/mL in well W1 microcosm and 3.88 Log_10_CFU/mL in well W2 microcosm over the same period. *V. cholera *decreased over time but surviving cells persisted for longer period in filtered-nonautoclaved water from well W1 (1.91 Log_10_CFU/mL) and well W2 (2.09 Log_10_CFU/mL). *Salmonella* sp. (poultry strain) did not follow the same survival pattern in well W2 microcosm as in well W1 microcosm.

*Salmonella* sp. and *V. cholerae* survived with little change in cell density in both autoclaved and filtered well water. While *Salmonella* sp. (human, pig, and water strains) maintained the same pattern of growth in filtered-autoclaved water from wells W1 and W2, *E. coli* progressively decreased its number to undetectable level (<1 Log_10_CFU/mL) after 2-3 days. *Salmonella* sp. (poultry strain) and *E. coli* did not follow the same pattern of growth in filtered-autoclaved water from wells W1 and W2. After the decrease to undetectable level (<1 Log_10_CFU/mL) at day 2, *E. coli* grew in filtered-autoclaved water from well W2 and reached 1.61 Log_10_CFU/mL after 3 days.

Of the six bacteria tested, *E. coli* had the highest decrease in viable counts in any of the microcosms, although the decrease in viable counts was slightly less pronounced in unfiltered-autoclaved water derived from well W2 than in the other test microcosms. 

### 3.2. Discussion

#### 3.2.1. Evaluation of Well Water Quality

*Physicochemical Parameters*. The physicochemical properties of water samples varied from one well point to another (Table 1). These fluctuations were also observed by several authors in the study of groundwater quality in some parts of the city of Yaoundé [13]. They are likely linked to differences in human population occupation, spatial heterogeneity of the soil of the region, and the variability of potential retention of microorganisms and chemicals by this soil.

Comparing the raw and treated well water samples, it seems that treatment leads to a decrease in the concentrations of the major elements. For instance, salinity and electrical conductivity reached respective minimum levels of 244 ppm and 491 *μ*S/cm in treated water. This may be related to the retention of suspended solids to filter or the modification of molecules structure. However, this decrease in concentration of minerals does not exceed the safe limits for human consumption according to WHO [14].

*Bacteriological Quality*. The results revealed that all investigated raw well water samples contain considerable numbers of heterotrophic bacteria, total coliforms (TC), faecal coliforms (FC), and faecal streptococci (FS) (Table 2) which are monitored as classical indicators of pollution and exceeding the permissible limit recommended by WHO. Several contemporary studies have shown that the presence of FCs and streptococci in groundwater is a frequent phenomenon. The presence of coliforms in drinking water has been regarded as an important marker of water safety [15]. The bacterial pollution of well water is mostly due to poorly constructed and located latrines, open rubbish dumps, and open drains [16]. Due to space limitations and lack of a proper drainage network, the traditional latrines system is extensively used in this area, and faecal matter from these latrines into the nearby wells might have contaminated the well water sources. There is a need to educate the public about the quality of their water sources and the importance of clean and healthy surroundings near water sources and to implement measures to prevent the contamination of water sources in the community.

Boiling and/or filtering water is advised until disinfection and retesting to confirm that the contamination has been eliminated.

#### 3.2.2. Survival and/or Growth of Bacteria in Well Water Microcosms

Treatment of well water by filtration and/or boiling is often presented as an adequate solution to compensate the lack of drinking water in poor communities in developing countries [17, 18]. Such treatment results in clean drinking water. However, secondary contamination of the treated water during storage at home could compromise this effort. This study assessed the ability of some faecal bacteria (*E. coli, Salmonella* spp., *and V. cholerae*) of various origins (human, animal, and well water) to survive and/or grow in well water treated by filtration and/or autoclaving.

During three days of water storage, faecal bacteria survive in different microcosms to varying degrees. The dynamics of bacterial abundances, in most cases, can be divided into two phases: a phase of relative stability or low decrease in bacterial abundances, followed by a phase of relative growth or bacterial inhibition (Figure 1). The first period is similar to a phase of bacterial adaptation to the organic matter available in trace in the water, in conditions offered by the storage. This phase lasts between 1 and 2 days of water storage.

The second phase is similar to bacterial latency or accelerated development of cells and is visible on the 2nd or 3rd day, reflecting the depletion of nutrients in the environment or good bacterial adaptation to new environmental conditions. Overall, the bacterial latency seems to depend on the origin and type of water as well as the bacterial strain. Microorganisms rapidly develop mechanisms to adapt to changes in their environmental conditions [19]. The results showed that the unfiltered-autoclaved well water better supports the survival of these organisms. Indeed, the organic substances dissolved in water in the absence of the native microflora increase the survival and growth of bacteria such as *Salmonella* spp. and* E. coli* [20]. The filtered water, autoclaved or not, is less favourable to the survival/growth of the bacteria tested. In this study, the content of suspended solids has not been determined. However, it is clear that water filtration reduces the amount of suspended solids. This would lead to ion binding to the filter and lower the level of mineralization. The low degree of mineralization increases the antibacterial activity of the environment [21]. Le Chevallier et al. [22] reported that the growth of heterotrophic bacteria would be limited in water containing less than 50 mg/L of assimilable organic carbon.

The filtered-nonautoclaved water is less favourable to bacterial survival/growth than filtered-autoclaved water. The filtration through the 0.2 *μ*m filter retained the great majority of autochthonous bacteria [23]. Autoclaving inactivates most of living organisms in the water treated, reducing the interactions between experimental bacteria inoculated and autochthonous microflora. Biotic factors are important factors of growth inhibition of *Salmonella* sp. and* E. coli*. These biotic factors are all made of predation by protozoa and other bacteria, phage lysis, and competition with autochthonous bacteria [24, 25]. According to Mary et al. [23], the potential survival of several species of allochthonous bacteria in filtered-nonautoclaved water is sometimes significantly reduced by the presence of “ultramicrocells” of autochthonous bacterial microflora, due to nutrient competition which would be exerted by bacteriolytic enzymes of membrane vesicles produced by native microflora.

Although no disinfectant has been added to the treated water, abundances of bacterial pathogens decreased during storage of household water out of reach of any source of secondary contamination (insects, dust, dirty hands, and soiled containers) [26]. The unfiltered-autoclaved water increases the chances of survival and growth for all bacteria, whereas filtered-nonautoclaved water seems to increase the possibility of negative interactions by competition for nutrients between microorganisms. The availability of organic carbon, higher in the unfiltered-autoclaved water, is the key factor of microbial growth control [27].

The survival of *E. coli* in all microcosms was relatively lower than that of all tested *Salmonella* spp. This result is in consonance with those of several previous studies [28]. In addition, *V. cholerae* has presented a particularly important survival. Several authors have shown that *V. cholerae* can grow in freshwater in vitro (concentrations between 2,9 × 10^5^ and 1,6 × 10^6^ cells/mL). In addition, *V. cholerae* is even able to grow in competition with microbial communities in the water [29].

Bacterial strains of *Salmonella* spp. from well water showed no specific higher growth/survival than clinical strains during storage of water. This confirms the role of populations in the contamination of the environment by the test strains.

The well water provided at source is unsafe for consumption with regard to microbial indicators. The absence of these bacteria in autoclaved and/or filtered well water indicates that filtration and boiling can be recommended if properly carried out. However, the survival and/or growth of some pathogenic bacteria in autoclaved and/or filtered well water, during storage at home, suggest the importance of water treatment and safe storage at household level. According to our results and taking into account the economical context of the region which is too poor, it is recommended that rather than discouraging the use of traditional latrines, users should be made aware of the importance of discarding them from water sources and maintaining them hygienically.

## Figures and Tables

**Table 1:** Mean (and standard deviation) values of some physicochemical parameters taken from wells and microcosms water samples.

Parameters	Wells							
	W1				W2			
	Raw water	Unfiltered-autoclaved	Filtered-nonautoclaved	Filtered-autoclaved	Raw water	Unfiltered-autoclaved	Filtered-nonautoclaved	Filtered-autoclaved
pH (CU)	6.85 (0.26)	7.32 (0.12)	7.20 (0.15)	7.18 (0.17)	7.52 (0.15)	7.68 (0.11)	7.71 (0.09)	7.78 (0.13)
Conductivity (*µ*S/cm)	626 (49.94)	567 (15.20)	510 (16.30)	491 (17.00)	1154 (96.77)	1164 (21.00)	1142 (18.00)	1129 (32.00)
TDS (mg/L)	435 (36.39)	395 (17.00)	364 (33.25)	343 (34.25)	819 (65.25)	800 (31.00)	797 (21.00)	768 (25.00)
Salinity (ppm)	315 (24.85)	282 (15.20)	267 (11.00)	244 (17.00)	586 (45.40)	582 (43.00)	563 (39.70)	557 (31.90)

Sample size (*n* = 4).

**Table 2:** Averages (and standard deviation) values of bacteriological parameters analyzed in each point of well water.

Sampling site	Bacterial abundances (CFU/100 mL)				
	HPC	Total coliforms	Faecal coliforms	*Escherichia coli *	Faecal streptococci
W1	3.45 × 10^6^ (5.0 × 10^4^)	1.83 × 10^4^ (8.19 × 10^2^)	9.33 × 10^3^ (3.21 × 10^2^)	3.53 × 10^3^ (5.8 × 10^1^)	3.57 × 10^2^ (3.8 × 10^1^)
W2	7.03 × 10^7^ (1.15 × 10^4^)	3.06 × 10^4^ (1.24 × 10^3^)	2.56 × 10^4^ (3.83 × 10^3^)	2.93 × 10^2^ (1.10 × 10^1^)	3.67 × 10^3^ (2.08 × 10^2^)

Sample size (*n* = 4).
